# Pathology, Practical Medicine, and Therapeutics

**Published:** 1837-07

**Authors:** 


### PATHOLOGY, PRACTICAL MEDICINE, AND THERAPEUTICS.

*On the Indications for the Use of Chlorine and Muriatic Acid Vapours in Diseases of the Air-Passages and Lungs.* By Professor ALBERS, of Bonn.

[IN the years 1829, 1830, and 1831, Professor Albers instituted a series of clinical experiments in the Medical Hospital at Bonn, on the effects of chlorine vapours in phthisis, chronic bronchitis, and chronic pneumonia; these experiments were repeated in his private practice, during the years 1832 and 1833, and derive much value from the care taken to establish the diagnosis in every case with sufficient accuracy, a point to which very little attention had been paid by preceding writers on the subject.]

The chlorine vapour was applied in the manner recommended by Murray; or, instead of exposing the patient to vapour strongly impregnated with chlorine, for the space of a few minutes at different times in the day, he was kept the whole day in a chamber filled with very weak chlorine vapour. The vapour was produced by boiling chloride of lime and then heating it in a large dish, or by sprinkling it with muriatic acid; sometimes it was generated by pouring sulphuric acid on culinary salt. The following cases exhibit the results of Dr. Albers's experience.

CASE I. A woman, aged forty-seven, of consumptive habit, and born of parents who died of consumption, was admitted into the hospital in January, 1830. Her symptoms were, cough of twelve months standing, progressive emaciation, hectic, great dyspnœa, copious expectoration of tubercular matter streaked with blood, sharp rapid pulse, anorexia, and constipation. She had pectoriloquy and mucous rhonchus under both clavicles; absence of the respiratory murmur at some spots, and bronchial respiration at others; respiration normal in the lower part of the right side only. There could be no doubt of the existence of phthisis in this case.

As soon as the hæmoptysis had ceased under the use of acetate of lead and nitre, the patient was exposed to the chlorine vapour for the space of fifteen minutes four times a day. On the third day, she complained of violent constriction of the chest, copious expectoration, and stitches in the side, and was obliged to omit the use of the vapour. Two days afterwards the experiment was again repeated, but had to be discontinued a second time from the same cause. The only effect observed, was a diminution in the frequency and hardness of the pulse. During the use of this remedy she had observed a mild nutritious diet. She died five months afterwards.

Case ii. A man, aged twenty-six, was admitted into the Medical Clinic in February 1831. His symptoms were, cough of two years standing, (said to have arisen after exposure to cold,) latterly increased, and accompanied by expectoration; fixed pain in a circumscribed spot between the fifth and sixth ribs on the right side, about three fingers' breadth from the sternum; occasional attacks of dyspnœa, ending in copious expectoration of clear viscid mucus mixed with firm globular masses streaked with blood; habit of body normal; slight emaciation. Over the spot already noticed, sonorous and sibilous rhonchi were alternately heard; on the left side, the respiratory murmur was nearly natural; on the right side, particularly at the upper part of the chest, it was feeble, and bronchophony was heard at the spot where the sibilous rhonchus was present. The diagnosis here recorded was "ulceration of the bronchial tubes, with a healthy state of the parenchyma of the lungs." Under the use of cupping, blisters, and expectorants, the pain disappeared, and the respiratory murmur in the right side became clear, but the cough and expectoration continued, with a slight sibilous rhonchus. The patient remained for a few minutes, four times a day, in a chamber abundantly filled with chlorine vapour. On the first day, he had violent constriction of the chest at each inspiration; on the second he was somewhat easier, but on the sixth his breathing was so much oppressed that he thought he should be suffocated, and the expectoration stopped. He was afterwards cured by other means.

Case iii. I. J., aged twenty-six, born of healthy parents, and without any sign of the scrofulous diathesis, except a few swollen glands in the neck, placed himself under the care of Dr. Albers in 1831. For the last six months he had laboured under cough, at first dry, but afterwards attended with expectoration of greyish, fetid, tubercular matter; he had pectoriloquy under the left clavicle, and the usual signs of tubercles in the second stage, but no pain in any part of the chest. After having used the ordinary remedies for a fortnight, he was kept the whole day in a chamber filled with weak chlorine vapour. He suffered considerably at first from constriction of the chest, although there was no diminution of the cough or expectoration, but subsequently bore the vapour extremely well, and remained in it for twenty-three days without any remarkable inconvenience. During this period there was no diminution of his symptoms; the expectoration became more copious, and lost its fetid odour, and pectoriloquy became developed under the right clavicle also. Shortly afterwards, he was attacked with colliquative diarrhœa and died. Dissection exhibited tubercles in all stages, and ulceration of the intestines.

Case iv. A man, aged forty-six, who had been two years under treatment in the medical clinic at Bonn, and who exhibited all the signs and symptoms of tubercular disease of the lungs in the third stage, was exposed to the chlorine vapour in the manner already described. At first, the inspiration of the vapour was distressing, but afterwards agreed very well with the patient, and he continued to use it for the space of five weeks without any intermission. The expectoration became more fluid and less fætid, the hectic paroxysms less regular, the pulse less excited, and the nights were passed more easily; the local symptoms, however, continued unchanged, and the patient sank two months afterwards under the usual colliquative diarrhœa.

Case v. A man, aged thirty-three, who had laboured for six months under cough with copious expectoration of thick yellowish matter, placed himself under the care of Dr. Albers. His symptoms were, dyspnœa, increased by motion; emaciation, occasional pains in the chest, and cough, which was so violent at night and in the morning that the patient was afraid of being suffocated. The stethoscopic signs were, bronchophony over the whole of the right side, bronchial respiration, and mucous rhonchus at various spots in the same side, and clear sound on percussion. His appetite and digestion were good, and he had no hereditary tendency to phthisis.—*Diagnosis:* Dilatation of the bronchial tubes.—The patient was exposed to a very weak vapour of chlorine, which he bore tolerably well for the first day; but on the second, he suffered great distress of respiration, and on the third the expectoration stopped, and his symptoms became so violent that he was obliged to give up the remedy. After eight days, during which the patient was brought back to his former state, a second experiment was made, but was followed by the same result; the sense of constriction and suffocation was so great, that the man could not endure it, and the use of the vapour was wholly abandoned. The patient died some time afterwards, and on opening the body, the lungs were found healthy, and the bronchial tubes generally dilated.

Case vi. A man, aged twenty-two, and previously healthy, was attacked in the winter of 1831, after hard labour, with violent pain in the right breast, increased during motion and inspiration, dyspnœa, and short cough, accompanied by scanty expectoration, at first of a dark brown, but afterwards of a bright red colour. He was blooded three times, had forty leeches applied to the right side of the chest, and used several mixtures with more or less relief. After some days, the cough increased, and he began to expectorate a fetid matter of a yellowish grey colour; his fever diminished, but the dyspnœa and sense of constriction in the chest remained, with occasional stitches in the side. Under these circumstances he was admitted into the medical hospital at Bonn, and presented the following train of symptoms:—Chest well formed, right subclavicular region somewhat less prominent than the left, and tender on pressure; respiratory murmur distinct in the whole of the left lung anteriorly and posteriorly; on the right side, it was absent in the subclavicular region; over the remainder of the right side it could be heard distinctly. When the respiratory murmur was absent, mucous and cavernous rhonchi were heard, and pectoriloquy, circumscribed to a small spot. Decubitus on the back or the left side brought on violent paroxysms of coughing; the expectoration was greenish yellow, very fætid, and occasionally streaked with blood; the patient had regular attacks of hectic every afternoon, his pulse was always hard and frequent, his strength greatly diminished, and he appeared to be considerably emaciated.—*Diagnosis:* an ulcerating vomica in the upper lobe of the right lung. Under the use of a mild diet, the constriction of the chest and bloody expectoration disappeared, and it was then determined to expose the patient for a few minutes each day to the vapour of chlorine. The first two turns, the patient was unable to remain in it longer than a minute or two, in consequence of the cough and sense of suffocation produced by it, but by degrees he began to bear it better, and at the end of three weeks was able to remain a quarter of an hour in the room six times a day. During this period, the fever diminished, the cough became less violent, the patient's appetite returned, and the night sweats disappeared; the expectoration however remained the same, except that it contained less mucus. The patient became now anxious to return home, and left the hospital. In 1835, Professor Albers heard from a physician in the patient's neighbourhood, that he had progressively improved after he left the Hospital, and was able to serve in the Landwehr without any further inconvenience.

Case vii. A girl, aged twenty, and born of healthy parents, was attacked, in November 1830, with febrile symptoms, accompanied by dyspnœa, flying pains in the whole of the left side of the chest, hard short cough, and bloody expectoration. Under the use of local and general depletion, the pains diminished in violence, but the cough became increased, and she continued to have copious expectoration of yellowish green matter, mixed with air bubbles, and streaked with blood. When seen by Dr. Albers, she seemed considerably emaciated, and exhibited indications of the scrofulous diathesis; but she had no fever, and her sleep and digestion were normal. Her pulse was hard, her cough violent night and morning, at other times less troublesome; expectoration copious, fetid, and of a greenish yellow colour, sinking in water; decubitus on the right side always produced severe paroxysms of cough. The left mammary and subclavicular regions were tender on pressure; at this spot, a distinct bubbling sound was heard, and bronchophony was slightly audible in the morning, but distinctly in the evening after copious expectoration. The respiratory murmur could scarcely be heard over the whole of the upper part of the right lung; in the left lung, the respiration was puerile.—*Diagnosis:* Ulcerating vomica, with inflammation of the surrounding pulmonary tissue. As the patient exhibited a great tendency to hæmoptysis, she was blooded, took nitre, and used an antiphlogistic diet, until the expectoration no longer shewed any streaks of blood. She was then exposed to the chlorine vapour, which, in the beginning, she could only bear for half a minute at a time, but subsequently was able to breathe it for an hour, three times a day, without inconvenience. Under this treatment the expectoration became diminished and lost its fetid odour; the cough improved, and the mucous rhonchus disappeared, but the pectoriloquy remained. After using the chlorine vapour for six weeks, the quantity of expectorated matter was scarcely one sixteenth of the original amount, she had hardly any cough, and the expectoration was quite free. She then went to the country and used a milk diet, and when examined again by Dr. Albers, in August, 1831, she was free from cough and expectoration, and capable of pursuing her ordinary employments. At the spot where pectoriloquy had formerly existed, there was still an indistinct bronchophony, and in the surrounding parts the respiratory murmur was very feeble. In other respects, the lung was quite natural.

The following are the conclusions deduced by Professor Albers from the results of his experience in numerous cases:

1. Chlorine acts as a stimulant, but when the remedy gets into the blood, its effects are antiphlogistic. Its effects as a local stimulant are observable in every one of the foregoing cases. It produces a tickling sensation in the eyes, nostrils, and throat, and sense of roughness and constriction in the chest. If the throat of an individual who has been exposed for a long time to the vapour of chlorine be examined, it will generally be found of a deeper red than natural. These and other facts are sufficient to prove that chlorine vapour operates as a local stimulant. It is on this account more remarkable that chlorine when it gets into the blood, exercises an influence of a directly opposite nature. It diminishes the frequency of the pulse, calms excitement, and produces effects which may be termed antiphlogistic. This difference of effect, however, before and after the remedy gets into the blood, is not peculiar to chlorine; it is common to many remedies of an antiphlogistic character, and is observed in nitre, tartar emetic, and many of the neutral salts. When there is no hæmoptysis or violent local irritation present, chlorine inhalations may be used in diseases of the lungs and air passages; its stimulant effect gradually diminishes, and after some time the mucous surfaces of the lung become less sensible to its exciting influence.

2. In tubercles of the lungs, in chronic catarrh, in chronic inflammation and ulceration of the bronchial mucous membrane, and in dilatation of the bronchi, chlorine vapour is of no service, and in most cases will not be borne, in consequence of the irritation it produces. On the other hand, it has a very salutary operation in pure ulceration of the lungs or vomica. This state, however, is not to be confounded with suppurating pneumonia, to which the use of chlorine vapour is not so applicable. How far patients labouring under disease of the lungs may be adapted for using this remedy cannot be determined; much will depend on general irritability and individual disposition, and the chlorine vapour should be always tried experimentally at first.

3. From the foregoing observations it appears, that chlorine vapour produces salutary effects in chronic ulcers of the lungs; this agrees with the results obtained in surgical practice from treating old ulcers with the solutions of chloride of soda and chloride of lime.

[The experience of Professor Albers coincides in some points with that of Dr. William Stokes, of Dublin. Dr. Stokes has always found chlorine inhalations prejudicial in phthisis, producing in all cases increase of bronchial irritation, dyspnœa, and arrest of the pulmonary secretion. In his trials of this remedy in gangrene of the lung, of which an interesting detail will be found in the fifth volume of the Dublin Hospital Reports, he has found it decidedly beneficial, correcting the fœtor of breath and expectoration, and therefore calculated to obviate, not only the local, but also the constitutional symptoms connected with such a state of the lung.]

*Hannoversche Annalen.* 1836.

---

### Professors Sebastian and Albers *on Tubercles.*

A valuable article on Tubercles has lately been published by Professor Sebastian in the Dutch periodical, *Tijdschrift voor natuurlike Geschiednis,* 2d Deel, 3d Stuck. We have not seen the original paper, but we subjoin a short notice of the Professor's opinions, as we find them stated by Dr. Albers, of Bonn, in an article contained in the Hannoverian Annals. Sebastian is of opinion that tubercles are deposited in the air-cells of the lungs, and not in the intercellular tissue; chiefly on the ground that the latter does not afford space sufficient for such deposition, and, secondly, because tubercles are never found in the interlobular cellular tissue. The question, whether hydatids are ever the cause of tubercles is negatived by Sebastian, as far as regards the lungs; but he is inclined to think that tubercles in the liver may owe their development to such a cause. Dr. Albers, however, has convinced himself that hydatids are never the cause of tubercles, either in the liver or in any other organ; but he admits that a deposition of tubercular matter may take place in the hydatid itself, where a predisposition to the tubercular diathesis exists. Both Sebastian and Albers agree that tubercles are never owing to degeneration of structure, in glandular or any other tissue; and the latter has frequently satisfied himself by microscopical investigations that tubercular infiltration arises from the deposition of an infinite number of small tubercles between the healthy fibres of an organ, and never to degeneration of structure.

Sebastian adheres to the opinion that tubercles are fluid when first deposited, chiefly because he believes that the vessels are incapable of secreting solid matter; miliary tubercles he regards as genuine tubercles, but Albers is disposed to consider this opinion as still open to objection. Sebastian considers the enlargement of tubercles as in most cases owing to aggregation, but he does not deny that in some cases it may be owing to a greater original secretion of tubercular matter. It is obvious that, during the period of softening, tubercles are often enlarged by aggregation; but the variable size of the crude tubercle must be owing either to a greater or less tubercular deposition, or to the nature of the tissue in which the tubercle is deposited. The latter cause has been advocated chiefly by Carswell and Schröder van der Kolk; but, as Sebastian maintains that the crude tubercle displays the same original conformation in whatever organ it may be deposited, he is inclined to ascribe to it a peculiar power of self-formation. This, he says, is more particularly the case with miliary tubercles, which, in every situation, display the same peculiar round shape. The variable consistency of tubercles is supposed to be owing to the greater or less absorption of the fluid of the tubercular matter, and not to depend, as Carswell thinks, upon the organ in which it is deposited. The latter cause may, however, be of some effect; for, supposing that tubercular matter, when secreted, is always of a like consistency, it must act as an irritant more in one organ than in another.

Sebastian subscribes to the now generally received opinion that tubercles are not supplied with blood-vessels, and he supports this view by beautifully injected preparations. His opinions regarding the softening of tubercles, and the enlargement of the tubercular cavity, do not appear to contain any thing novel. Some doubts still exist regarding the manner in which the chalky concretions, which are observed to follow the resolution of tubercles in their first stage, are formed. Sebastian thinks that the tubercle may be directly changed into this chalky matter; a supposition which Albers considers as highly improbable; and maintains, on the contrary, that the tubercle must first be absorbed, and the chalky matter afterwards deposited. Sebastian agrees with Andral and Laennec in considering the cicatrization of tubercular cavities as possible, and even not unfrequent: with this opinion Albers by no means agrees, and chiefly on the ground that tubercles are rarely found, in such cases, either in the lungs or in other organs; when such cicatrices exist, he ascribes them to the obliteration of pueumonic abscesses, in which supposition his own experience, he says, amply supports him.

*Hannoversche Annalen.* Erster Band, drittes Heft.

---

*On the Indications for the Use of Moxa, with Cases.* By DR. SADLER. (From the Archives of the Corresponding Medical Society of St. Petersburg.)

THE moxas employed by Dr. Sadler are about half an inch in diameter, and three quarters of an inch in height. They are composed of a nucleus formed of the pith of the sun-flower (Helianthus annuus), wrapped in layers of cotton of various thickness, and surrounded with an external envelope of thin muslin; both the latter are previously steeped in a solution of nitre. They are held, while burning, by means of two long hair pins, the legs of which are slightly bent, in order to accommodate them to the shape of the moxa; and when the latter is burned down to the place where it is held by the first hair pin, it can be seized with the other, and retained in its proper situation. The moxa must be allowed to act for a considerable time: by observing this rule, the pain after the operation is always considerably less than where the application has been continued only for a brief period. The burnt part requires no treatment for the first few days; it is insensible and dry. Larrey states that moistening the part with spirit of ammonia prevents suppuration: this however is not the fact; suppuration always takes place, and is often very copious. Dr. Sadler never uses more than one moxa at a time, except in cases of violent sciatica, in which he sometimes applies one over the glutei, and another over the most painful spot of the thigh or calf. Except in one instance, which appears somewhat doubtful, he has never seen any secondary bad effects from the use of the moxa. When used in febrile affections, it generally produces an increase of the fever on the second day. In painful affections, the relief afforded is often instantaneous, and lasts many days. Generally speaking, however, it ceases about the fifth day, and it is on this day that he usually repeats the application, and so on every fifth day until the requisite number have been used. He has never used more than eight, and in most cases not more than three. In one case he used twenty-four, but then they were employed at considerable intervals during the course of two years, and the moxas were only about one third of the usual size.

The general effects of moxa, on which the indications for its use depend, are divided by Dr. Sadler into, 1, anti-erethistic; 2, purely dynamic; 3, excitant; 4, revulsive; 5, reparative. To illustrate these, he gives the following cases, to which are appended a few more by Dr. Busch and Dr. Wrangell.

I. *Anti-erithistic.* 1*st Case.* Miss B., was attacked with violent symptoms, which left her medical attendant in doubt whether they were the precursory symptoms of measles, or connected with a spasmodic affection of the chest to which she was subject. A small quantity of blood was abstracted on the third and fourth day, and on the fifth, a moderately diffused eruption of measles appeared, accompanied by violent gastro-enteric irritation. The vomiting and purging continued in spite of every means, and on the sixth day, the pulse began to sink, and the extremities became cold. A moderately sized moxa, applied over the epigastrium, speedily arrested the vomiting, and removed the threatened danger. The child convalesced slowly, but recovered completely.

2*d Case.* E. von der L., aged twenty-eight, of delicate constitution, and who had been frequently ill during the winter of 1833-4, consulted Dr. Sadler on the 18th of July, 1834. He presented the usual symptoms of dyspepsia in an aggravated form, namely, weakness, irritability, emaciation, loss of sleep, foul tongue, salivation, anorexia, diarrhœa, heartburn, pain in the chest, and dry cough. He was ordered to use a milk diet, and prescribed the nitrate of potash with laurel water in mucilaginous decoctions; but as the symptoms continued progressive, and the patient became worse, Dr. Sadler applied a moxa over the epigastrium, on the 13th of August. This produced considerable relief, and was repeated on the 17th. On the 19th, the patient felt greatly improved, and his appetite was returning. On the 29th, he was able to dispense with medicine altogether. He recovered rapidly, and is now quite well.

II. *Purely dynamic: 1st Case.* Herr von St., a strong healthy man, aged fifty, had laboured for several months under a violent pain in the thigh-bone, supposed to have arisen from exposure to cold. No trace of disease could be discovered on examination, and during the day the patient felt quite well, with the exception of a slight degree of languor; but at night when in bed, he was attacked at an uncertain hour with the most intolerable pain, which lasted until morning. He had tried various remedies for several months, but without effect. Six moxas, two over the glutei, and four over the most painful part of the thigh, completely relieved him. In the winter of 1832, he had a second attack, produced also by exposure to cold, and was cured again by the application of three moxas.

*2d Case.* A riding-master, aged forty, who had fractured his leg and thigh, and dislocated the hip-joint by a fall from his horse, consulted Dr. Sadler in December, 1835. He complained of violent pain in the hip, thigh, and calf, which frequently lasted from two to four weeks, totally preventing him from sleeping, and then became for some time much milder. His health was somewhat impaired from loss of sleep, but in other respects his constitution was good. He was completely cured by four moxas, two on the hip, one on the middle of the thigh, and one on the calf. He bore the application of the first two on the hip without much suffering, but the two following ones caused excessive pain.

IV. *Revulsive: 1st Case.* A shoemaker, aged forty, of emaciated cachectic appearance, consulted Dr. Sadler on the 13th of February, 1833, affected with copious expectoration of fetid purulent matter, great emaciation, and colliquative sweats. In addition to the internal use of acetate of lead and opium, a moxa was applied on the 16th over the most painful part of the left side of the chest. A second was applied on the 20th, but he refused to submit to a third, which was to be applied five days afterwards. His improvement was slow, but evident; the expectoration became diminished and lost its fetid smell. Iceland moss, milk diet, and a residence in the country completed his cure.

*2d Case.* A married woman, aged twenty-two, of delicate make but sound constitution, caught cold in September 1835, while menstruating. The catamenia were arrested, and she was attacked with violent inflammatory fever, which yielded after some time to general and local depletion, but the patient remained weak and emaciated, and complained of constant pain in the lower part of the abdomen. On examination, a large fluctuating tumour was discovered in the situation of the right ovary. A large moxa was applied over the central part of the tumour, and bark with a nutritious diet prescribed. Five days afterwards, a second was applied over the fundus uteri, and the use of tonics and aperients was continued. Under this treatment, the swelling diminished greatly, the patient began to improve in health and strength, and left the hospital on the fortieth day with the moxas still suppurating. The catamenia appeared again on the 20th of December, and soon afterwards the patient was able to resume her occupation as a washerwoman. The affected ovary never returned to the normal size, but it ceased to give any inconvenience whatever.

V. *Reparative.* Mademoiselle J., aged twenty-two, of full habit, and subject to some irregularity of the catamenia, had laboured for two years under an affection of the stomach, accompanied by constant burning pain in the epigastrium, particularly after taking food, vomiting, salivation, and costiveness. During this period she had used various remedies without success, and consulted Dr. Sadler in 1832, who, having tried the usual means without any result, proposed the use of moxa. A moxa was applied over the region of the stomach, which produced so much relief that the patient came herself five times to have it repeated. Her recovery was complete and permanent.

The first two of the following cases are given by Dr. Busch, the third by Dr. Wrangell.

CASE I. A man, aged fifty, of intemperate habits, who had been labouring for three weeks under mucous fever accompanied by diarrhœa, was suddenly attacked with inflammation of the right lung. He was leeched twice, had cupping-glasses applied several times, and a purulent discharge was kept up for a considerable time by means of blistering ointment. Notwithstanding these measures, the pneumonia ran on to suppuration, the diarrhœa continued, and hectic fever set in, attended by great emaciation and sinking of the pulse. After the application of six moxas at intervals of six or eight days, the hectic vanished, and the expectoration ceased to have a purulent appearance. The cure was completed by the use of senega, laurel water, &c. but it was nearly half a year before the patient could leave the house. Dr. Busch observes that he has never witnessed febrile symptoms after the use of moxa, but that he has seen the suppuration continue for several months, although the part was bathed with spirit ammoniæ after the operation.

CASE II. A young man of healthy constitution, was seized with complete paralysis of the lower extremities, after an attack of the prevailing epidemic fever in the spring of 1833. He had also a sensation of heat in the region of the sacrum, and violent pain increased by slight pressure, which compelled him to lie constantly on his belly, and tortured him day and night without any remission. Leeches, cupping-glasses, sinapisms, blisters, &c. were frequently employed without effect. After the application of two moxas, the feeling of heat disappeared, the motion of the affected limbs gradually returned, and two months afterwards the patient left the hospital quite well. Suppuration continued for a long time.

CASE III. A boy, aged five years, was attacked with hæmaturia; but as he appeared otherwise well, his mother took no notice of the occurrence. Some days afterwards he began to complain of pains in the thighs while walking, and his mother brought him to Dr. Wrangell. Oily and mucilaginous remedies were at first employed, but without benefit; the same results attended the use of cold applications over the loins, uva ursi, lycopodium, &c. and the urine began to exhibit a purulent deposit. A moxa was then applied over the region of the right kidney, and after some time, another on the opposite side. Under this treatment, all the unfavorable symptoms yielded, and the child got perfectly well.

*Zeitschrift für die gesammte Medicin.* Band iii. Heft ii. and iii.

---

*Contest between Scarlatina and Small-pox.* By DR. GLEHN.
(From the Transactions of the St. Petersburg Medical Society.)

ON the 25th of November, 1834, scarlatina and small-pox being at that time epidemic, a young sea officer was seized with vomiting without any apparent cause. He felt better on the 26th, but on the 27th complained of headach, vertigo, and languor, with quick pulse, loaded tongue, suffusion of the eyes, and alternate rigors and flushes of heat. His symptoms were increased to an extreme degree of violence on the 28th, and on the 29th a marbled eruption of scarlatina, of dark red colour, made its appearance on his face, neck, breast, and upper extremities; along with this, there were some solitary pimples observed on his forehead and face. The velum and uvula were inflamed, and he had considerable difficulty of swallowing. Next day, the scarlet eruption grew pale, and disappeared, while, on the other hand, the pimples became more numerous and distinct, and were speedily recognized as belonging to the variety of small-pox termed varioloid. The variolous eruption afterwards extended over the whole body. From the moment the scarlatina disappeared, all the bad symptoms ceased, and the disease ran through its course with great mildness. A case of the same description is mentioned by Hufeland in his observations on the small-pox and vaccine at Weimar, in 1798. In his case also the small-pox obtained the mastery.

*Zeitschrift für die Gesammte Medicin. November,* 1836.

*On the Treatment of Ileus with Belladonna Clysters.* By Dr. Wagner, District Physician at Schlieben.

The following cases are given in illustration of the treatment recommended by Hanius.

Case i. On the 21st of April, Dr. Lohrenz of Schönewalde was called to visit a man, aged twenty-three, who had been complaining, since the 19th, of violent pains in the umbilical region. The pains came on periodically, and were greatly exacerbated by pressure, so that the patient screamed out when touched. He had incessant retching, his belly was hard and tense, and he had been several days without an alvine evacuation. Venesection, leeches, enemata, and various other external and internal remedies, were employed without any effect; his symptoms increased in intensity, and on the 22d, he had subsultus, syncope, and vomiting of feculent matter. His belly was tympanitic hard and painful, his bowels obstinately costive, his pulse scarcely to be felt, his anxiety intolerable, and his body covered with a clammy sweat. Under these circumstances, Dr. Lohrenz had recourse to clysters of belladonna. One half of the lavement was first injected; and unlike the other enemata, which were almost immediately rejected, this was retained, and had a marked effect in calming the violence of the patient's symptoms. His countenance became more cheerful, and his abdomen softer, but the pupils became greatly dilated. Half an hour afterwards, the second half was injected, and produced the most decided improvement. It was speedily followed by copious evacuations from the bowels, the pulse rose, the pain and vomiting ceased, and next morning the patient felt quite restored, and has not had since that time any return of his complaint.

Case ii. On the 4th of June, Dr. Wagner was called to see a labourer's wife, aged forty, of spare habit, but otherwise robust and healthy. The patient complained of a violent cutting sensation in the bowels, with obstinate costiveness and incessant vomiting. She had had repeated attacks of the same description before, but much milder, and of brief duration. On examination, he found a hernial tumour in the right groin, about the size of a walnut, and so excessively tender on pressure, that she could not bear the slightest touch. The belly was tympanitic and tender, the pulse small and rapid, the face pale, the body moderately warm. A large venesection was premised, and all the usual internal remedies (except quicksilver) tried without any effect; clysters of all kinds were employed, but proved equally ineffectual. The patient refused to submit to a second venesection or the application of leeches, and rejected altogether the proposal of an operation. On the 5th, all her symptoms were increased; her thirst was excessive, and she had fecal vomiting, with suppression of urine. In this state of things Dr. Wagner had recourse to the belladonna clysters. He infused a drachm of the root of belladonna, and an ounce of chamomile flowers (he does not state how long) in twelve ounces of water, and divided the infusion into three parts. The first part was administered by himself as soon as it was cold, and produced very remarkable effects. The nausea and vomiting instantly ceased, and half an hour afterwards, the belly was soft, without much tenderness on pressure, and the hernial tumour much less tense, though still painful. None of the secondary bad effects of belladonna were observed. On visiting the patient at noon, he found her quite easy and contented, but labouring under dilatation of the pupils. She told him that she had been threatened with a repetition of the attack about half an hour before, but that she had stopped it *by drinking a few spoonsful of the clyster mixture.* In the evening, when seen by Dr. Wagner, she complained of a return of the abdominal pain and tension, and as there was no indication of the secondary effects of the belladonna, except some dilatation of the pupil, he administered the remainder of the infusion. The patient passed a quiet night, with the exception of some troublesome dreams, and, on the following morning, the abdominal symptoms were mild and inconsiderable, except that the hernial sac remained extremely tender on pressure, and the incarcerated portion of intestine could not be replaced. At noon, the soreness and tension of the belly increased again, and as no alvine evacuation had as yet taken place, and there were no apparent bad consequences from the belladonna, Dr. Wagner repeated the infusion as before. The first dose produced the usual tranquillizing effect, but no further change; and as the constitutional effects of the remedy were limited to some increase in the dilatation of the pupils, with unpleasant dreams, he administered the second portion, and, towards evening, the third and last.

On the morning of the 7th, the hernial tumour had disappeared, loud borborygmi were heard in the abdomen, and large evacuations of offensive fæces took place, but the patient, after having been annoyed the whole night with frightful dreams, was suddenly seized with such furious delirium that it required several strong men to hold her. Her eye was fixed and sparkling, the pupils excessively dilated, the conjunctiva injected, the cheeks of a fiery red, the pulse small, rapid, and scarcely to be felt, *deglutition unimpeded.* She saw nothing but strange phantoms, which she sought to drive away by abuse and threats, and searched for concealed enemies under her bedding, clothes, and furniture. She believed herself perfectly well, wished to resume her domestic labours, pulled on her clothes with furious violence, and would have rushed out of the house had she not been held by force. Dr. Wagner ordered enemata of vinegar, (which were followed by copious evacuations,) and gave vinegar with strong coffee internally, of which the patient drank large quantities with much desire. Cold lotions were applied to the head, and the limbs were washed with vinegar, an operation which the patient herself performed with apparent satisfaction, washing herself with vinegar from head to foot. This state of things continued until the morning of the 8th, when the patient became rational and composed, but complained of flashes of light and various other optical phantasms, with a sense of great weight and pressure in the head, and a general feeling of soreness and exhaustion, particularly in the feet. She recollected distinctly everything she had said and done during the preceding day and night, and said that the horrible phantoms by which she was incessantly surrounded had compelled her to act and speak in the manner she had done. On the 9th, she complained of nothing but weakness, which soon disappeared, and she recovered rapidly without any farther unpleasant symptoms.

CASE III. On the 3d of July, a smith, aged fifty-nine, was attacked with enterodynia, vomiting, tympanitic swelling of the abdomen, and constipation. Dr. Wagner was called to see the patient, and found an incarcerated hernia of the left groin, about the size of a hen's egg, and extremely sore to the touch. All external and internal remedies, repeated local and general bleeding, and frictions over the abdomen with extract. belladonnæ and ol. hyoscyami, proved wholly ineffectual. Every thing was instantly vomited up, and the clysters were immediately returned. As the patient would not submit to an operation, Dr. Wagner threw up an enema, composed of a scruple of the belladonna herb, and half an ounce of chamomile flowers, in four ounces of water, which arrested the vomiting immediately, and produced such a diminution of the pain, that the patient was able to enjoy several hours' sleep. The abdominal symptoms, however, returned every six or eight hours, and were four times allayed by the use of the same enema. On the 5th, return of the pain and tenderness; Dr. Wagner was afraid to have recourse to the belladonna, as, in addition to great dilatation of the pupils, frightful dreams, sinking and acceleration of the pulse, and dryness of the tongue, had taken place; and he prevailed on the patient, after much entreaty, to submit to the operation. This was performed successfully by Dr. Weistand, on the 6th, and in fourteen days the patient was quite well.

CASE IV. On the 5th of July, Dr. Wagner was called to a woman, aged forty-seven, who was said to have been labouring for two days under violent pains in the abdomen, obstinate constipation, and incessant vomiting. On making an examination, he found an incarcerated femoral hernia of the right side, about the size of a small walnut, and excessively tender to the touch; diffused abdominal tenderness, and tympanitic distention. Bleeding, leeching, frictions over the abdomen with belladonna and hyosciamus, and various other remedies, were employed without any effect; and the symptoms assumed a very alarming character. As the patient refused to submit to an operation, Dr. Wagner had recourse to the belladonna clysters, which produced the usual tranquillizing effects, but the hernia remained irreducible, and the patient began to exhibit some of the symptoms of poisoning, as dilatation of the pupils, sparkling of the eyes, a fiery red colour of the cheeks, and acceleration of pulse. Blood was now drawn from the arm, small doses of calomel and laxative salts given internally, and the belladonna clysters continued, until six lavements (each composed of ℈i. of belladonna to ℥iv. of water) were used. The hernia, however, remained irreducible, and as the patient would not submit to an operation, Dr. Wagner discontinued his visits on the 8th. On the 9th, however, the greater part of the hernial tumour had disappeared, the patient had several copious stools, and in the course of two days, found herself quite well.

[The foregoing cases show that belladonna, like tobacco, is a remedy of great efficacy in subduing symptoms of ileus connected with incarcerated hernia. It has also the advantage of relieving pain, without substituting for it the horrible sickness and sinking of the vital powers, which results from the use of tobacco. Two facts, however, connected with the history of belladonna, will always tend to diminish its applicability; namely, its tendency to accumulate in the system and then explode with fearful violence, and the well known fact, that its specific powers vary in a remarkable degree according to the place in which it grows.]

*Journal der Pr. Heilkunde. August,* 1836.

---

*Remarkable Case of Pica.* By Dr. Von Vogel, of Rostock.

Among the persons who spent the bathing season at Doberan, last summer, was a foreign lady, who had been affected for some years with an unnatural appetite for charcoal. She was thirty-two years old, and had been married ten years. Her first three labours terminated naturally; she then had a miscarriage, and during her pregnancy laboured under salivation. Shortly afterwards, she conceived again, and was a second time attacked with salivation, which continued during the first half of the period of utero-gestation. Towards the end of her pregnancy, she suffered greatly from abdominal derangement, hæmorrhoids, and spasmodic affections of the bladder, all of which disappeared after her confinement. With the exception of an hysteric habit her health was generally good, except that she was troubled with acidity of stomach, uneasiness, and nausea. She had also frequent attacks of lippitudo, her urine was turbid and sabulous, and all her family were subject to gout and hæmorrhoids.

For the last year and a half she has never been free from the appetite for charcoal, which manifested itself for the first time in her third pregnancy, during which she had frequently used charcoal for acidity of stomach, by the advice of her physician. Its influence upon her spirits is most remarkable; whenever she is sad, uneasy, or squeamish, she flies to charcoal for relief. It always tranquillizes and cheers her, and the greater her distress is, the more ravenous is her appetite for charcoal. After eating it, the bad taste in her mouth, the sense of acidity, and heartburn, completely disappear.

When the monthly period is about to set in, the appetite for charcoal becomes very sharp, so that the latter indicates the approach of the former. During this time, the charcoal is most agreeable to her palate; she carries it constantly by her, and consumes several papers of it in the day. She gives a preference to hard-wood charcoal, and suffers no inconvenience from using it, as far as she can observe, but constipation. She has used the waters of Carlsbad, and sea-bathing at Doberan, with considerable benefit to her general health, but without any change in the appetite for charcoal.

*Hufeland and Osann's Journal.* lxxxiii. Band., iii. Stück.

---

*Rare Case of Spontaneous Human Combustion.* By Dr. Patrix.

Therese Lemaitre, aged sixty years, the mother of many infants, suffered from no other infirmity than some chronic ulcers upon the legs, which rendered her lame.

She was exceedingly stout. She kept herself constantly intoxicated by the abuse of wine. Returning home on the 15th December, 1836, through a street which she knew imperfectly, she is supposed to have made a false step and fallen, and was there found extended, resembling more the remains of a mummy than any other cadaverous form. This woman was accustomed to clothe herself with two or three garments, and to return home in the evening, guided by a lantern suspended from the girdle of her robe. In her habitual state of intoxication, it is supposed that a false step having thrown her into a pool of mud, seven or eight inches deep, the lantern was broken and forthwith set fire to the greasy garments, thus establishing a centre of combustion.

The body was found slightly inclining to the left: the fall of the intestines to this side also indicated this position. The cranium and face were dried up by the combustion, but the right side of the face was consumed, including the osseous substance. The right side of the chest was hardened and deeply cracked. The right arm was untouched and stretched out, as if demanding succour. The muscles upon the lateral and posterior part of the same side of the body were pale and partly burnt. The combustion was more severe according as the body was examined from above downwards. No trace of the sex remained. The tissues of the thigh were destroyed. The os femoris was bare. The leg had been in part preserved by the bandage and dressing of the ulcers. On the left side of the body the ravages were more severe. The femur was covered by pale muscles, in part roasted; no trace of the integuments remained.

Upon attempting to lift the body, the right femur was grasped but immediately pulverized, and when the trunk was raised a crackling was heard as if the skeleton were broken. *La Lancette Française. December*, 1836.

---

*Tobacco in Tetanus.* By Dr. CAVENUE, of Martinique.

M. CAVENUE, practising in a country where the occurrence of tetanus is very frequent, has had recourse to the use of enemata of infusions of tobacco in many cases, and with great success. He has recorded various instances of recovery after this treatment; sufficient, indeed, to call renewed attention to a mode of practice which, although already employed in this disease, is less known and appreciated than it deserves to be. Of the successful cases recorded in this paper, two are of traumatic tetanus: one following suddenly on the use of a cold bath during profuse perspiration; and another, subsequent to the bite of a serpent, which is almost always fatal in its effects. M. Cavenue recommends the use of tobacco generally in the bites of poisonous serpents; and the same means may be worthy of employment in cases of hydrophobia. This is rendered more probable from one case which is related, of a young negro, who became the subject of trismus from a wound produced by a fragment of glass. He presented hydrophobic symptoms, and recovered after an antiphlogistic treatment, to which was added, with the most evident advantage, the employment of a tobacco lavement. Nothing is said as to the quantity of the vegetable which should be administered. M. Cavenau appears in one case to have used a quantity which would be enormous in the healthy state. But the strength of the remedy must, probably, be always proportioned to the intensity of the disease. In serious cases, from one to two drachms would probably be sufficient, as in the Enema Tabaci of the London Pharmacopœia.

*Bulletin de l'Academie Royale de Médecine,* No. V. 1836.

---

*Observations on the Vaccine Pustule, made during an Epidemic of Small-pox.* By M. DUCROS, Jun.

AT Marseilles, during an epidemic of small-pox, several hundred persons who had been vaccinated died, and considerable doubt was expressed regarding the importance of the discovery of Jenner. From several observations which M. Ducros made, it apears that the more the number of vaccine pustules are multiplied, the greater the power of the constitution to resist small-pox. Forty girls were admitted into the hospital at Marseilles with confluent small-pox, and the majority presented but one pustule in each arm. From this M. D. is convinced that one or two pustules are insufficient to preserve individuals from the disease. In his practice he vaccinates in several places in both arms, and often in the inferior extremities, so as to create a febrile action at the period of the eruption.

He thinks the vaccine virus should be renewed every year. For thirty years the virus had not been renewed at Marseilles, and he conceives that the ravages committed by the epidemic with which they were visited may be attributed to this circumstance. Indeed, the generality of physicians had convinced themselves of the truth of this remark, by using this year vaccine matter from England, which produced a much better vaccine pustule, with a more marked inflammatory areola.

*La Lancette Française. November*, 1836.

---

*Use of Chlorine Inhalations in acute and chronic Bronchitis, and in Bronchorrhœa.* By M. A. Toulmouche, of Rennes.

The greater number of the experiments, the inferences from which are here related, were made during a period of four years and a half, in a "Maison de Detention," where pulmonary catarrhs are very common. The majority of the patients have borne very well the first impression of the chlorine; and all have become capable of employing it, by gradually accustoming themselves to it. With the fewest exceptions,—such as where great irritability and oppression existed,—the chlorine was employed in every case which bore the name of pulmonary catarrh, acute or chronic, inflammatory or pituitous. Its sensible effect is to change the quality of the bronchial secretion, to diminish its quantity, and finally to put a stop to it.

The result of the use of chlorine in 228 females is recorded in this paper.

Of these 228, 141 were affected with acute, and 65 with chronic, bronchitis; 17 of which latter were double, 4 complicated with pulmonary emphysema, and 22 with phthisis. Of the 141 acute cases, 51 were cured in from five to six days; 33 in from seven to ten; 29 in two or three days, and 21 in from eleven to fifteen. The greater number were thus cured in from five to eight days; the smaller in from eleven to fifteen; a result much superior to that which is obtained by the ordinary means. Of the 65 cases of chronic bronchitis, 16 were cured in from twenty-one to ten days; 15 in from eleven to ten; 13 in from two to ten; and one only in eighty-eight days. The average of cures requires, therefore, from sixteen to thirty days; and two-thirds of the patients recovered in from five to twenty or twenty-five days. This is regarded as a period of treatment two or three times shorter than that which is commonly employed. The ordinary dose is from thirty to forty drops, commencing with ten, and gradually increasing. M. Toulmouche has employed as many as 180 drops, but he does not consider that the value of the remedy is thus increased.

*Bulletin de l'Academie Royale de Médecine,* No. 6. 1836.

